# Substance P concentrations in the blood plasma and serum of adult cattle and calves during different painful procedures and conditions – a systematic review

**DOI:** 10.1186/s12917-022-03304-6

**Published:** 2022-06-18

**Authors:** Theresa Tschoner, Melanie Feist

**Affiliations:** Clinic for Ruminants With Ambulatory and Herd Health Services at the Centre for Clinical Veterinary Medicine, Ludwig Maximilian University of Munich, Sonnenstrasse 16, 85764 Oberschleissheim, Germany

**Keywords:** Analgesia, Castration, Dehorning, Pain assessment, Pain management, Surgery

## Abstract

**Background:**

Pain in cattle is a major welfare problem, as cattle mask their pain. Subjective and objective parameters to assess pain in cattle have been described. Among the objective parameters to evaluate pain in cattle is substance P (SP). SP is a neurotransmitter, which is involved in the processing of noxious information to the brain; it seems to be a more objective indicator for nociception than cortisol, which has long been used as a biomarker for pain and stress in cattle. The objective of this systematic review was to assess the existing literature about SP during painful procedures, conditions, and diseases in cattle in form of a systematic review.

**Results:**

Following the PRISMA statement, 36 out of 236 studies were included in this systematic review. Study design, grouping, age and weight of animals, processing of blood samples for the assessment of SP, and results were heterogenous. The largest number of studies originated from the United States of America and Canada and were published in 2018. A higher number of studies were done on calves (69.4%, *n* = 25) compared with adult cattle (30.6%, *n* = 11). Most studies were done to assess SP concentrations after administration of analgesics prior to husbandry procedures in calves.

**Conclusions:**

There is a manageable number of studies assessing SP concentrations during painful procedures, conditions, and diseases in cattle. SP seems to be a suitable biomarker for nociception in cattle, but results of research work are heterogenous, and SP concentrations of calves and adult cattle differ throughout studies. Basic research work is missing and is needed to assess factors others than nociception which might influence the SP concentrations in the blood plasma.

**Supplementary Information:**

The online version contains supplementary material available at 10.1186/s12917-022-03304-6.

## Background

In cattle, the recognition and therefore the management of pain is a major welfare problem [1, 2]. This is caused by the fact that cattle, as prey animals, strongly mask pain-associated behavior [3]. Different parameters for the assessment of pain in adult cattle and calves have been described; subjective pain assessment (such as ethograms [4], Numerical Rating [1, 2], Visual Analogue [5], or Facial Grimace [6] Scales) is always dependent on the observer’s experience and attitude [3], contrary to the use objective pain parameters (e.g. cortisol [7, 8], mechanical nociceptive threshold [9], accelerometers [10] and pedometers [11], and infrared thermography [12]). Among others, Substance P (SP) is considered an objective biomarker for pain.

As a neurotransmitter (tachykinin), SP is involved in the processing of noxious sensory information to the brain [13]. SP, which is composed of 11 amino acids (Arg-Pro-Lys-Pro-Gln-Gln-Phe-Phe-Gly-Leu-MetNH_2)_ [14–16] is synthesized as a prepropeptide in ribosomes and transported to the nerve ends via axons. Following a (thermal, mechanic, or chemical) noxious stimulus, SP is released from the neurons of the spinal ganglion and can be found in afferent neurons of the dorsal horn of the spinal cord, in cells of the dorsal ganglion, and in the dorsal roots of spinal nerves [16]. SP is primarily released from C-fibers, and its release is described to be slow [17].

SP was first used as a pain marker in bovine medicine in 2008 by [8]. The authors showed that the plasma SP concentrations increased significantly in castrated compared with sham-castrated calves, contrary to the cortisol concentrations, which increased in both groups [8]. Since then, various studies investigating the SP concentrations in adult cattle and calves during different (painful) procedures and conditions have been published. However, SP concentrations vary throughout the literature [8], and high variations between calves have been described [8, 18]. Additionally, Dockweiler et al. (2013) found an age difference in SP concentrations in calves [19]. Also, there are varying reports about the relationship between SP and procedures related to pain such as surgical castration [20] or disbudding [21], with no difference in SP concentrations between control animals and animals which had been treated with analgesic drugs.

Reviews have been published about pain assessment in cattle [22–26], but to this day, there is no systematic review about the use of SP as a biomarker for pain.

Therefore, the objective of the present paper was to describe and compare SP concentrations in adult cattle and calves associated with different (painful) procedures, conditions, and diseases. The aim of this review is to be a contribution to the current knowledge by giving an overview of literature concerning research about SP in bovine, and to identify and outline areas of lack of knowledge.

## Material and methods

### Search strategy and criteria for selection

The present systematic review was done following the study protocol for PRISMA-P (Preferred Reporting Items for Systematic Reviews and Meta-Analysis protocols) as published by Shamseer et al. [27] (Additional file 1) and described by [28]. The literature search was conducted on the 28^th^ of September 2021 and was limited to peer-reviewed articles in English and German. For this systematic review, the following 3 electronic scientific literature databases were used: PubMed (including MEDLINE), Web of Science, and Agricola. The main elements of this review were Cattle, Substance P, and Pain, and the same code was used for all three databases; the population search terms were (cattle OR cow OR calves OR bull OR steer), and the outcome search terms were (“substance P”) and (pain* OR nocicept*). For this systematic review, calves were defined as cattle ≤ the age of 12 months.

### Selection of studies

According to the search items stated above, studies of all designs and different languages describing the evaluation of Substance P during various procedures in cattle and calves were admitted into the study selection. Studies with English or German titles were included in the search, whereas studies in other languages and studies which were not accessible in any way were omitted. Following the exclusion of all duplicate studies, two authors (TT, MF) independently evaluated all titles of the remaining publications to check if the eligibility criteria (studies about pain assessment in cattle or calves) were fulfilled. Titles including other species than cattle were excluded, as well as reviews. The abstracts of the remaining studies were screened by two authors (TT, MF) for the eligibility criteria, and if a study appeared to be eligible, the full text was retrieved. Full texts were screened by one author (TT) and were included in the systematic review, if the following questions could be answered with yes, as described by [29]:

1. Is the study population either cattle or calves?
2. Is SP used as a biomarker for pain/nociception?
3. Are animals undergoing a painful procedure (such as castration, dehorning, etc.) or condition/disease?
4. Is the article peer-reviewed?

In cases of uncertainty whether a study should be included, the second author (MF) was consulted to decide upon the decision.

### Data extraction

The first author (TT) screened all full texts and extracted all data regarding the author, year of publication, number of animals and grouping, timing, and processing of samples, and results (concentrations of SP).

## Results

### Findings: demographic

**Fig. 1:**
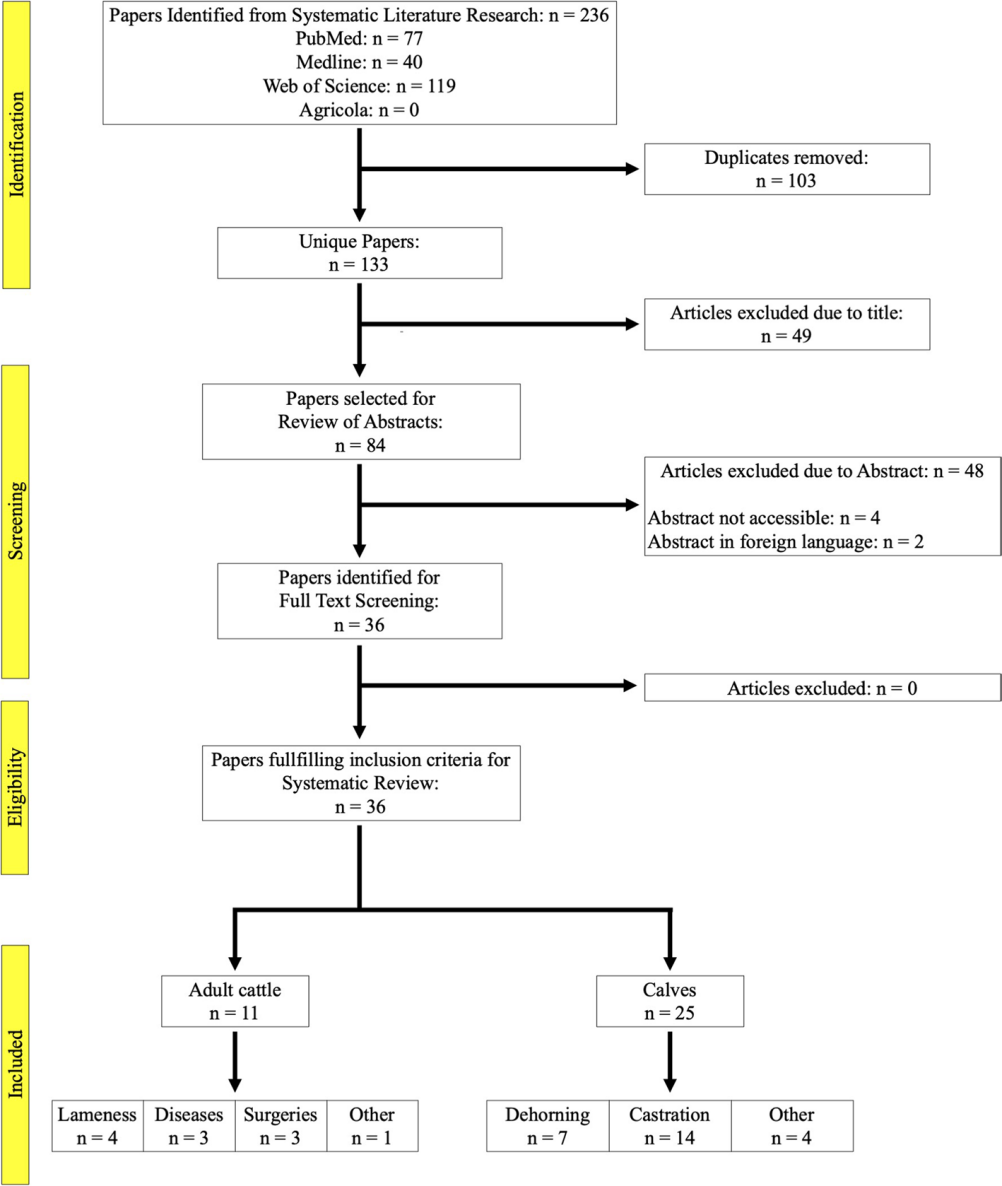
PRIMSA (Preferred Reporting Items for Systematic Reviews and Meta-Analysis) flow chart of the literature search and the selection of references during the review process for the evaluation of Substance P concentrations during different painful procedures, conditions, and diseases in adult cattle and calves

**Fig. 2:**
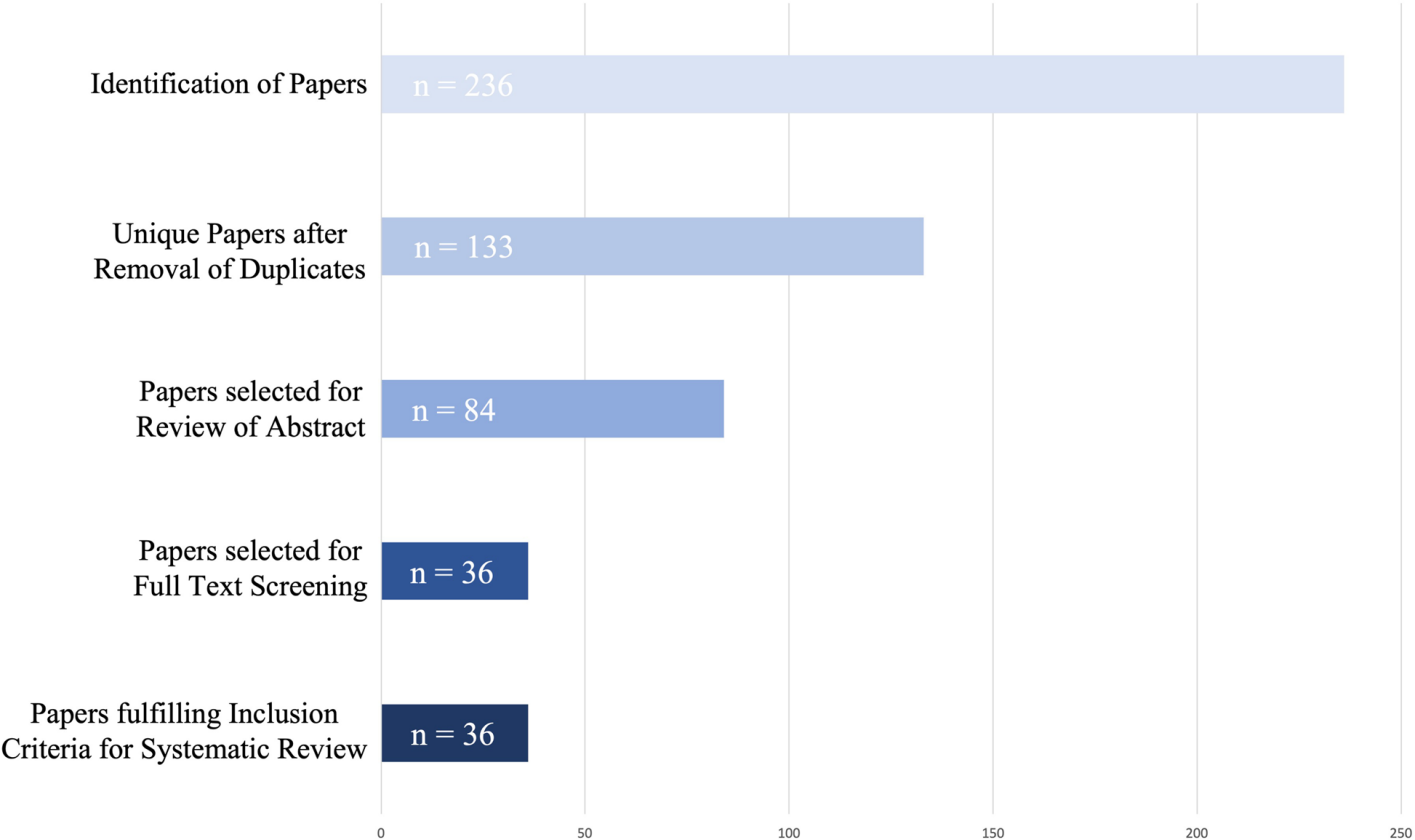
Publication number diagram for the selection of studies for a systematic review for the evaluation of Substance P concentrations during different painful procedures, conditions, and diseases in adult cattle and calves. Number of studies was reduced from 236 identified studies to 36 studies included in this systematic review (*n* = 11 for adult cattle and *n* = 25 for calves)

**Fig. 3:**
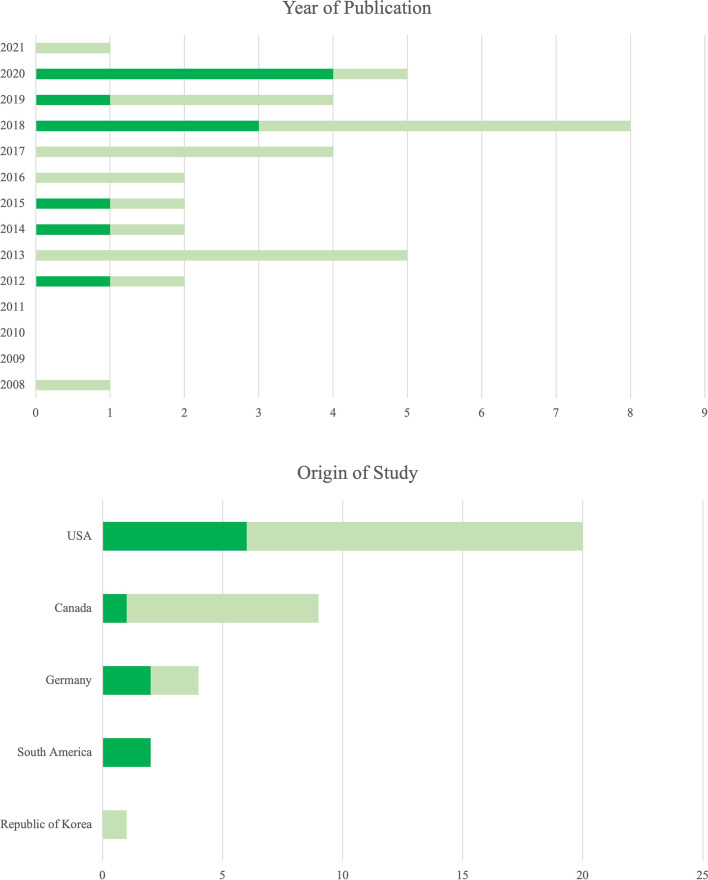
Demographic overview of range of publication year and origin of studies (country) in references about Substance P concentrations during painful procedures, conditions, and diseases in adult cattle (dark green) and calves (light green). The largest number of studies was published in 2018, and most studies originated from the United States of America (USA) or Canada

The literature search of the three databases resulted in a pool of 236 references; of these, 133 remained after deletion of all duplicate titles. A total of 49 references was excluded after screening of title, resulting in 84 references for screening of the abstract. Out of these references, 48 were excluded due to the reference not being eligible for the systematic review. Of these, 4 abstracts were not accessible, one was in French and one in Chinese. A PRISMA flow chart presenting an overview of the literature search and study selection is given in Fig. 1, and a publication number diagram is presented in Fig. 2. In total, we included 36 studies into the systematic review. With 69.4% (*n* = 25), most studies were conducted on calves, compared with 30,6% (*n* = 11) on cattle. Range of publication year was from 2008 to 2021 (Fig. 3), and studies were conducted in the United States of America (USA), Canada, Germany, South America, and the Republic of Korea (Fig. 2). The distribution of painful procedures and conditions/diseases in calves and cattle is given in Table 1.

**Table 1:** Distribution of painful procedures and conditions in 36 references used to evaluate the Substance P concentrations as a biomarker for pain in the blood plasma and serum of calves and adult cattle. For the present systematic review, calves were defined as cattle ≤ the age of 12 months

Procedure/Condition	Total number of articles
**Calves**	25
Castration	14
Dehorning	7
Other	4
**Adult Cattle**	11
(Induced) Lameness	4
Diseases/Conditions	3
Surgeries/Procedures	3
Other	1
**Total Number of Articles**	36

### Findings: material and methods

**Fig. 4:**
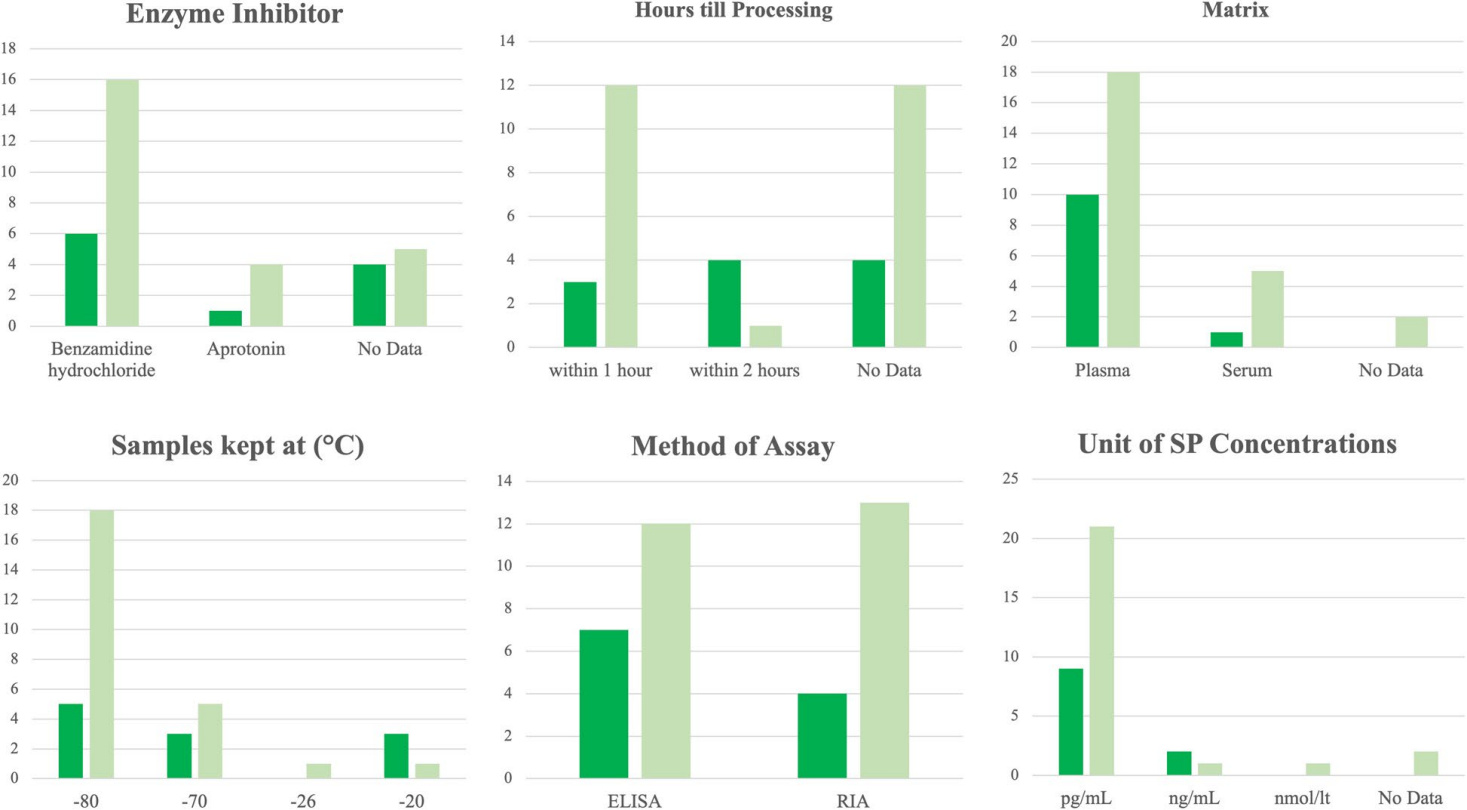
Summary of processing of samples as described in the Material and Methods section of studies in cattle (*n* = 11, dark green) and calves (*n* = 25, light green) evaluating Substance P (SP)  concentrations during different painful procedures, conditions, and diseases. In one study, SP concentrations were given as pg/mL and ng/mL; for this analysis, ng/mL (in-text) was used. Competitive Immunoassays (*n* = 3 in cattle and *n* = 5 in calves) and Enzyme Immunoassays (*n* = 2 in calves) were considered as ELISA. In one study, samples were kept at -18 °C, until transportation to the authors’ clinic, where samples were then kept at -80 °C; these were included as stored at -80 °C. One study described SP concentrations in blood as well as in saliva samples

Processing of blood samples (*n* = 36 studies) and saliva samples (*n* = 1 study) as described in Material and Methods was heterogenous. A summary of inhibitor used to keep SP from degradation, hours until processing and centrifugation of blood samples, matrix (blood plasma or serum) used, temperature at which samples were kept until analysis, method as assaying, and unit used for the evaluation of SP concentrations is presented in Fig. 4. Samples were kept on ice until processing or cooled/refrigerated in 54.1% (*n* = 6) and 18.2% (*n* = 2) in cattle, and 52% (*n* = 13) and 4% (*n* = 1) of calves, respectively.

In 27.3% (*n* = 3) of studies in cattle, and 44% (*n* = 11) studies in calves, there was no information about storing of samples for the determination of SP until processing and centrifugation.

### Findings: funding

Funding information was provided for 84% (*n* = 21) and 81.8% (*n* = 9) studies in calves and cattle, respectively (Additional file 2).

### Findings: calves

A total of 25 studies evaluating SP concentrations in calves during painful procedures, conditions, or diseases were identified. SP concentrations were evaluated for dehorning (28%, *n* = 7), castration (56%, *n* = 14), and other procedures and conditions/diseases (16%, *n* = 4). Year of publication, authors, grouping of animals (including administration of local anesthesia (LA) prior to painful procedures), time of blood sampling, extractable SP concentrations, and overall results are presented in Table 2.

**Table 2:** Summary of publication year, reference (Ref.), painful procedures/condition/disease, grouping and age of animals, sampling times, Substance P (SP) concentrations and conclusion of 25 studies using SP for the evaluation of nociception in calves. The administration of local anesthesia (LA) is given in the same column as the grouping of animals. In 32% (*n* = 8), no extractable data was presented

Year	Ref	Procedure/Disease with/without LA	Grouping	Age/Weight	Sampling Times and Assay Platform	SP Concentrations	Conclusions
**Castration**							
**2008**	[8]	Surgical Castration	- Surgical Castration (*n* = 5) - Sham Castration (*n* = 5) - no LA	4 to 6 months	- Baseline (24 and 12 h before procedure) - immediately after procedure - 10, 20, 30 and 45 min after procedure - 1, 1.5, 2, 2.5, 4 and 4 h after procedure - Competitive Immunoassay	Mean and SEM plasma SP Concentrations Surgical Castration C_min_ 303.98 ± 119.73 pg/mL C_max_ 888.92 ± 235.44 pg/mL Sham Castration C_min_ 88.68 ± 23.93 pg/mL C_max_ 691.38 ± 71.83 pg/ml	Castrated calves showed significantly (*p* = 0.042) greater mean plasma SP concentrations for all time points after castration or simulated castration than sham castrated calves. Clear between- and within- calf variations of SP throughout the study period were observed
**2013**	[19]	Band castration Cut-and-Clamp Cut-and-Pull	- CONT (control, *n* = 20) - BAND (*n* = 18) - CLAMP (*n* = 20) - PULL (*n* = 18) - no LA	8 weeks (n = 40) 6 months (n = 40)	- Baseline - 60, 120, 240, 480, and 5760 min after castration - Competitive Immunoassay	No extractable numerical data	SP concentrations differed significantly (*p* = 0.01) by age; regardless of the method of castration, 6-months-old calves showed higher SP concentrations relative to 8-week-old calves
	[30]	Band Castration	- BAND (n = 7) - BAND-MEL (*n* = 7, castration and meloxicam) - SHAM (*n* = 7, sham castration) - no LA	300.8 ± 4.96 kg	- Day 0 - Day 1 - Day 7 of trial - Competitive Immunoassay	LSM plasma SP Concentrations BAND, Day 0 143.05 pg/mL BAND, Day 1 167.24 pg/mL BAND-MEL, Day 0 158.69 pg/mL BAND-MEL, Day 1 159.66 pg/mL SHAM, Day 0 166.36 pg/mL SHAM, Day 1 151.45 pg/mL	There was no difference in plasma SP concentrations across treatments; meloxicam was administered on days -1, 0, and 1 in a dose of 1.0, 0.5, and 0.5 mg/kg BW respectively
**2014**	[31]	Surgical Castration	- Flunixine (*n* = 24) - Placebo (*n* = 24) - ring block of 2% lidocaine for both groups	25 ± 2 days	- directly before treatment with NSAID/Placebo - days 1, 2, 3, 7, 14, 21, 28, 35, and 49 - RIA	Mean and SE plasma SP Concentrations Flunixine 34 ± 1.1 pg/mL Saline 34 ± 1.1 pg/mL Baseline 41 ± 1.2 pg/mL Day 3 34 ± 1.2 pg/mL Day 21 30 ± 1.2 pg/mL	An effect of day on serum SP concentrations was observed (*p* < 0.001). SP concentrations were highest at baseline, dropped by day 3, and leveled out by day 21. The application of flunixin meglumine (1.1 mg/kg BW IV) had no effect on the serum SP concentrations; also, there was no interaction between drug an day
**2016**	[10]	Band Castration Surgical Castration	for both castration methods each - Meloxicam (*n* = 15) - Control (*n* = 15) - no LA	4 to 5 months	- Day -2 - 5, 24, 48, and 72 h following castration - Competitive Enzyme Immunoassay	Mean and SE plasma SP Concentrations Day -1 Band, Meloxicam 243.9 ± 16.4 pg/mL Band, Control 268.2 ± 15.6 pg/mL Surgical, Meloxicam 249.8 ± 7.8 pg/mL Surgical, Control 244.5 ± 11.5 pg/mL Day 0 Band, Meloxicam 243.7 ± 13.9 pg/mL Band, Control 340.5 ± 23.0 pg/mL Surgical, Meloxicam 267.9 ± 11.2 pg/mL Surgical, Control 314.7 ± 13.4 pg/mL Day 1 Band, Meloxicam 261.8 ± 15.5 pg/mL Band, Control 335.5 ± 23.8 pg/mL Surgical, Meloxicam 260.6 ± 8.1 pg/mL Surgical, Control 304.4 ± 17.0 pg/mL Day 2 Band, Meloxicam 251.6 ± 14.9 pg/mL Band, Control 295.2 ± 16.3 pg/mL Surgical, Meloxicam 273.2 ± 10.4 pg/mL Surgical, Control 301.1 ± 17.0 pg/mL Day 3 Band, Meloxicam 253.7 ± 12.7 pg/mL Band, Control 264.9 ± 16.4 pg/mL Surgical, Meloxicam 333.7 ± 11.2 pg/mL Surgical, Control 334.8 ± 5.3 pg/mL	Plasma SP concentrations were significantly (*p* < 0.05) higher in control compared with meloxicam (1 mg/kg BW orally) treated animals, both on day 0 and day 1 and both for band as well as surgically castrated calves
**2017**	[32]	Band Castration Knife Castration	- CT (sham castration) - BA (band castration) - KN (knife castration) - no LA	12 calves each per group 1 week (*n* = 36) 2 months (*n* = 36) 4 months (*n* = 36)	- Baseline (D-1), immediately before castration - weekly afterwards until end of trial (= sloughing off of testicles of banded calves) - RIA	SP concentrations 1 week old Control 60.6 pg/mL Band Castration 63.9 pg/mL Knife Castration 62.0 pg/mL 2 months old Control 80.1 pg/mL Band Castration 76.2 pg/mL Knife Castration 81.0 pg/mL 4 months old Control 103.3 pg/mL Band Castration 100.3 pg/mL Knife Castration 100.0 pg/mL	There was no effect of treatment on SP concentrations in 1-week, 2-months, and 4-months old calves
	[33]	Band Castration Knife Castration	- CT (sham castration) - BA (band castration) - KN (knife castration) - no LA	12 calves each per group 1 week (*n* = 36) 2 months (*n* = 36) 4 months (*n* = 36)	- Baseline (D-1) - T0, 60, and 120 Minutes after castration - 7 days (D7) after castration - RIA	LSM serum SP Concentrations Day 0 and 7 after castration 1 week old: Control 92.6 pg/mL Band Castration 108.7 pg/mL Knife Castration 100.6 pg/mL 2 months old: Control 73.5 pg/mL Band Castration 70.1 pg/mL Knife Castration 66.8 pg/mL 4 months old: Control 102.9 pg/mL Band Castration 101.8 pg/mL Knife Castration 102.5 pg/mL 0, 60, 120 min after castration 1 week old: Control 72.1 pg/mL Band Castration 69.8 pg/mL Knife Castration 68.0 pg/mL 2 months old: Control 72.2 pg/mL Band Castration 70.6 pg/mL Knife Castration 68.9 pg/mL 4 months old: Control 103.5 pg/mL Band Castration 101.5 pg/mL Knife Castration 101.1 pg/mL	There was no treatment or interaction effect for SP in 1-week-old calves No treatment differences were seen for SP following castration in 2-months and 4-months-old calves
	[5]	Knife Castration	- 6H (NSAID 6 h prior), *n* = 11) - 3H (NSAID 3 h prior, *n* = 12) - 0H (NSAID 0 h prior, *n* = 11) - no LA	7 to 8 months	-D-7, D-5, D-2, D-1 before castration - immediately before castration (T0) - 30, 60, 120 and 240 min, after castration - 1, 2, 5, 7, 14, 21, and 28 days after castration - RIA	No extractable numerical data	There was no treatment or interaction effects for SP on the day of castration, but an overall increase in SP concentrations (*p* < 0.01) On days 1 to 28 after castration, there was a treatment x time interaction for SP (*p* = 0.01), with 6H and 3H calves (which received 0.5 mg/kg BW meloxicam SC 6 or 3 h prior to surgery, respectively) showing higher SP concentrations than 0H calves on day 1 after castration; 5 days following castration, SP concentrations tended to be higher in 3H compared with 6H calves
**2018**	[20]	Surgical Castration	- CAST + FLU (flunixin meglumine, *n* = 8) - CAST + PLBO (placebo, *n* = 8) - SHAM + PLBO (placebo, *n* = 8) - no LA	9 months	- Baseline on the morning of experiment - 1, 2, 4, 6, 8, 12, 24, 48, and 72 h after treatment application - RIA	No extractable numerical data	Following castration, there was no effect of topical flunixin meglumine (3.33 mg/kg BW) on SP concentrations. Also, there was no time effect or treatment by time interaction between the groups
	[34]	Band Castration Knife Castration	- CT (control, *n* = 24) - BA (band, *n* = 24) - KN (knife, *n* = 24) and in each group - NM (placebo, *n* = 36) - M (Meloxicam, *n* = 36) - no LA	7 to 8 days	- Day -1 - T0, 60, 90, and 120 min after castration - day 1, 2, 3, and 7 after castration - RIA	LSM serum SP Concentrations Minutes after castration Control, NM97.1 pg/mL Control, M 103.1 Band, NM 101.7 pg/mL Band, M 93.4 pg/mL Knife, NM 102.7 pg/mL Knife, M 99.5 pg/mL Days after castration Control, NM 92.3 pg/mL Control, M 99.1 pg/mL Band, NM 100.2 pg/mL Band, M 91.7 pg/mL Knife, NM 99.1 pg/mL Knife, M 90.1 pg/mL	There was a trend (*p* = 0.09) for SP concentrations to be higher in NM compared with M (meloxicam at 0.5 mg/kg SC) calves 120 min after castration. Also, SP concentrations were higher (*p* = 0.04) on day 7, and tended to be higher (*p* = 0.08) on day 3 after castration in NM than in M calves There was also a trend (*p* = 0.06) for SP concentrations to be higher in CT-M, BA-NM, KN-NM calves than BA-M and KN-M calves
	[35]	Knife Castration Branding	- CT (SHAM, *n* = 23) - KN (knife, *n* = 24) - BK (branding and knife, *n* = 24) and in each group - NM (placebo, *n* = 36) - M (Meloxicam, n = 36) - no LA	67 to 87 days	- 24 h (Day -1) before castration - immediately before (T0) castration - 60, 90, 120, 180 min after castration - day 1, 2, 3, and 7 after castration -RIA	LSM serum SP Concentrations Minutes after castration Control 81.8 pg/mL Knife, NM 80.1 pg/mL Knife, M 79.4 pg/mL Branding + Knife, NM 82.6 pg/mL Branding + Knife, M 70.0 pg/mL Days after castration Control 82.2 pg/mL Knife, NM 78.7 pg/mL Knife, M 75.8 pg/mL Branding + Knife, NM 84.5 pg/mL Branding + Knife, M 81.4 pg/mL	There was no effect of procedure of medication (Meloxicam, 0.5 mg/kg SC) for SP at any time after the procedure
	[36]	Surgical Castration	- NC-NLF (no castration, no analgesia, *n* = 10) - NC-LF (no castration, analgesia, *n* = 10) - C-NLF (castration, no analgesia, *n* = 10) - C-LF (castration, analgesia, *n* = 10) -ring block with 2% lidocaine hydrochloride for LF treatments	6.3 ± 0.09 months	- Immediately before castration - 0.5 and 6 h after castration - 1, 3, and 7 days after castration - ELISA	Plasma SP Concentrations 6 h following castration C-NLF 3.09 ng/mL NC-NLF 0.74 ng/mL Otherwise, no extractable numerical data	SP concentrations did not differ between groups 30 min after castration; at 6 h after castration, SP concentrations were significantly (*p* = 0.03) higher in C-NLF compared with C-LF (Castration with 12 ml lidocaine and 0.5 mg/kg BW flunixin meglumine) (3.09 ng/mL and 0.74 ng/mL respectively); SP concentrations returned to baseline values from day 1 on
**2019**	[37]	Knife Castration	- PO (Meloxicam, 1 mg/kg—BW orally, *n* = 12) - SC (Meloxicam, 0.5 mg/kg—BW SC, *n* = 11) - no LA	7 to 8 months	- Day -2 and -1 before castration - T0, and 30, 60, 90, 120, 150, and 240 min after castration - day 1, 2, 3, 5, 7, 10, 14, 21, and 29 after castration - RIA	LSM serum SP Concentrations PO 83.0 pg/mL SC 78.7 pg/mL	SP concentrations were higher (*p* ≤ 0.05) in PO compared with SC calves
**2021**	[38]	Surgical Castration	- SHAM (castration), followed by - CAST (24 h later) - no LA	6 weeks (*n* = 10) 3 months (*n* = 10) 6 months (*n* = 10)	- Immediately prior to both procedures (Time 0) - 1, 2, 4, 8, and 12 h after the procedures - Competitive Immunoassay	No extractable numerical data	At later recovery times, SP concentrations were lower in CAST compared with SHAM. Younger calves (6 weeks old) showed lower SP concentrations in CAST than in SHAM (*p* = 0.0174)
**Dehorning**							
**2012**	[39]	Scoop Dehorning	- Meloxicam (*n* = 6) - Placebo (*n* = 6) - no LA	16 to 20 weeks	- Baseline (prior to drug or placebo administration) - 5, 10, 15, 29, 30, and 60 min afterwards - 6, 22, 30, 45, and 52 h afterwards - ELISA	Mean and SEM plasma SP concentrations Placebo 114.70 ± 30.84 pg/mL Meloxicam 71.36 ± 20.84 pg/mL	Mean SP concentrations were significantly (*p* = 0.038) lower in meloxicam (0.5 mg/kg IV) treated compared with control calves. Plasma SP concentrations were estimated to be 0.5 less in the presence than in the absence of meloxicam treatment
**2013**	[40]	Cautery Dehorning	- MEL-PRE (NSAID pre-OP, *n* = 10) - MEL-POST (NSAID post-OP, *n* = 10) - CONT (control, *n* = 10) - cornual nerve block with 2% lidocaine hydrochloride for all groups	8 to 10 weeks	- 2 h before procedure (baseline) - 5, 30, 60, 120, 240, 360, 480, and 720 min after dehorning - Competitive Immunoassay	No extractable numerical data	At 120 min after dehorning, calves which received meloxicam (1 mg/kg orally) had significantly (*p* = 0.039) lower SP concentrations than control calves
	[41]	Scoop dehorning	- CONT (placebo, *n* = 8) - MEL (meloxicam, *n* = 8) - GBP (gabapentin, *n* = 8) - MEL + GBP (*n* = 8) - FLU (flunixin meglumine, *n* = 8) - cornual nerve block with 2% lidocaine hydrochloride for all groups	6 months	- Baseline (-10 min) before dehorning - 5 min after dehorning - 0.5, 1, 2, 4, 6, 8, and 12 h after dehorning - Competitive Immunoassay	Mean and SD plasma SP concentrations Calves not treated with Analgesia 137.29 ± 42.97 pg/mL Calves treated with Analgesia 63.35 ± 21.25 pg/mL	No differences between treatment groups (Placebo, Meloxicam (1 mg/kg) orally, Gabapentin capsules (15 mg/kg) orally, Meloxicam and Gabapentin orally, or Flunixine meglumine (2.2 mg/kg) IV) were found. Mean plasma SP concentrations were significantly (*p* = 0.02) lower in calves treated with analgesics compared with control calves
**2015**	[21]	Cautery Disbudding	- FIROCOXIB (NSAID, *n* = 10) - PLACEBO (*n* = 10) - cornual nerve block with 2% lidocaine hydrochloride	32.9 ± 3.9 days	- Baseline (1.5 h prior to dehorning) - 15 and 30 min, after dehorning - 1, 2, 4, 6, 8, 10, 12, 24, 48, 72, and 96 h after dehorning - RIA	LSM and SE of plasma SP concentrations FIROCOXIB 22.7 ± 0.7 pg/mL PLACEBO 20.8 ± 0.4 pg/mL	SP concentrations were not different between placebo and firocoxib (0.5 mg/kg orally) treated calves. There was no effect of time or time x treatment in the 96 h sampling period
**2016**	[42]	Cautery Dehorning	- SHAM (*n* = 10) - PO (NSAID orally, *n* = 10) - SQ (NSAID SC, *n* = 10) - PLCBO (placebo, *n* = 10) - cornual nerve block with 2% lidocaine hydrochloride	50.9 ± 5.3 days	- Baseline (1 h prior to drug administration) - 0.5, 0.75, 1, 2, 4, 6, 8, 10, 12, 24, 48, 72, and 96 h after dehorning - RIA	Mean plasma SP Concentrations with 95% CI Control PLCBO 17.0 pg/mL (14.8 – 19.5) SHAM 16.4 pg/mL (14.3 – 18.9) Carprofen PO 17.0 pg/mL (14.8 – 19.5) SQ 16.3 pg/mL (14.2 – 18.07)	No effect of treatment (carprofen, 1.4 mg/kg BW, either orally or SC), time, or time and treatment on SP concentrations. SP concentrations were greater (0.11 ± 0.039 pg/mL) in female than male calves (*p* = 0.005)
**2017**	[43]	Cautery Dehorning	- DH-FLU (dehorning and NSAID, *n* = 8) - SHAM-FLU (sham dehorning and NSAID, *n* = 8) - DH-PLBO (dehorning and placebo, *n* = 8) - no LA	6 to 8 weeks	- Baseline on the morning of experiment - 1, 2, 4, 12, 24, and 48 h after treatment application - RIA	Mean plasma SP Concentrations with 95% CI DH-FLU 103.5 pg/mL (92.7 – 114.4) SHAM-FLU (99.7 pg/mL (94.3 – 105.2) DH-PLBO 104.6 (96.2 – 113.1)	No effect of treament with topical flunixine meglumine (3.33 mg/kg) for SP concentrations
**2019**	[44]	Dehorning with Caustic Paste	- M1 (NSAID followed by placebo 24 h later, *n* = 15) - M2 (NSAID twice in 24 h, *n* = 15) - CONTROL (*n* = 16) - SHAM (sham disbudding, *n* = 15) - no LA	3 days	- Baseline (5 min prior to disbudding) - 24, 48, 72, and 96 h after disbudding - RIA	LSM plasma SP Concentrations Control 164.47 pg/mL Sham 198.53 pg/mL M1 144.50 pg/mL M2 144.74 pg/mL	Plasma SP concentrations not different between plasma SP concentrations between CONTROL and M2 (45 mg Meloxicam orally 24 h apart) than SHAM or M1 (45 mg Meloxicam orally, followed by placebo). Calves in SHAM had significantly (*p* < 0.0001) higher plasma SP concentrations
**Other**							
2013	[45]	Infection with *Mannheimia haemolytica* i	- MH (infected calves, *n* = 10) - CN (control, *n* = 8) - no LA	240.0 ± 13.1 kg	- Before challenge (D0) - 12 h after inoculation - day 1, 2, 3, 7, and 9 after inoculation - Immunoassay Kit (ELISA)	No extractable numerical data	There was a significant (*p* < 0.05) interaction between treatment group and trial day for SP concentrations. SP concentrations were significantly increased in MH compared with CT calves on D0.5 and decreased to average concentrations on D7
2018	[18]	Umbilical Surgery	- CON (meloxicam treated calves, *n* = 10) - MET (metamizole and meloxicam treated calves, *n* = 11) - no LA	37 ± 8 days	- Baseline (-1 h) before surgery - 5, 15, 30, 45, 90, 150, and 510 min after start of surgery (skin incision) - ELISA	Median plasma SP Concentrations Baseline CON 690.0 pg/mL (lower quartile 497.0 pg/mL; upper quartile 1301.1 pg/mL) MET 560.3 pg/ml (lower quartile 328.7 pg/mL; upper quartile 572.6 pg/mL CON T + 5 986.8 pg/mL T + 30 1217.2 pg/mL MET T + 60 541.1 pg/mL T + 150 555.6 pg/mL T + 510: 547.5 pg/ mL	Plasma SP concentrations were lower in MET (40 mg/kg BW metamizole IV and 0.5 mg/kg BW meloxicam IV) compared with CON (0.5 mg/kg BW meloxicam IV) at all times during and after surgery. In CON, plasma SP concentrations were significantly different from baselines at T5 (*p* = 0.027), T30 (*p* = 0.006) and T90 (*p* = 0.02). Calves of CON did not reach baseline plasma SP concentrations during the trial period
2019	[46]	Assisted Calving	- Meloxicam (*n* = 17) - Placebo (*n* = 16)	Newborn	- Birth (within 10 min of delivery) - 1, 4, 24 h and 7 and 10 days after delivery - RIA	No extractable numerical data	There was no significant difference between placebo and meloxicam (0.5 mg/kg BW SC) treated calves for SP concentrations over the 24-h period
2020	[47]	Tail Docking	- A (Amputation, *n* = 8) - K (Control, *n* = 8) - no LA	8 to 10 weeks	- D-2 to Day 2 at 11:10 am daily - D0 at 08:10 AM prior to tail docking via rubber ring - ELISA	No extractable numerical data	There was a trend (*p* = 0.087) for a difference between A and K for the mean SP concentrations. After differentiation of values after tail docking and handling, there was no difference in SP concentrations between groups

*BW* Bodyweight, *IV* Intravenously, *OP* Surgery, *SC* Subcutaneously, *RIA* Radioimmunoassay, *ELISA* Enzyme Linked Immunosorbent Essay

### Castration

Most studies using SP to evaluate pain during painful procedures in calves were done using castration as a painful stimulus (56%, *n* = 14). Study design was heterogenous (surgical/knife castration: 50% (*n* = 7), band castration: 7.1% (*n* = 1), band and knife castration: 28.6% (*n* = 4), band, cut-and-clamp, and cut-and-pull: 7.1% (*n* = 1), knife castration and branding: 7.1% (*n* = 1)), as was grouping of animals, and findings of the different studies. Coetzee et al. (2008) showed that plasma SP concentrations were significantly (*p* = 0.042) higher in surgical compared with sham-castrated calves [8]. After surgical castration, SP concentration only leveled out after 21 days [31]. According to Meléndez et al. (2017), an overall increase (*p* < 0.01) of SP concentrations was observed after surgical castration [5]. Also, there is an effect of administration of analgesics [36], as well as timing [5], and form of application [37] of NSAIDs on SP concentrations after surgical castration. When comparing surgical, band, and sham castration, there was no effect of treatment on calves of different age groups [32, 33]. Administration of meloxicam resulted in significantly (*p* < 0.05) [10] and by trend (*p* = 0.06) [34] lower SP concentrations in band and surgically castrated compared with control calves. Contrary to that, one reference stated that there was no effect of treatment (band castration, band castration an administration of meloxicam, or sham castration) on SP concentrations in calves [30]. All of the above-mentioned studies were performed without the administration of a LA.

### Dehorning

Evaluation of SP concentrations during and after dehorning was described for cautery and scoop dehorning and dehorning with a caustic paste (*n* = 4, *n* = 2, and *n* = 1, respectively). Study design was heterogenous, with variable grouping of animals and treatment with different NSAIDs. Contrary to one study stating that SP concentrations in dehorned calves were significantly (*p* = 0.039) lower in calves treated with meloxicam orally compared to untreated control calves (following a cornual nerve block for both groups) [40], other studies found that neither the administration of oral firocoxib (following a cornual nerve block for both groups) [21], nor carprofen (orally or subcutaneously, following a cornual nerve block for both groups) [42], or topical flunixin meglumine (no administration of LA) [43], had an effect on SP concentrations during or after cautery dehorning. Differences in results were also observed concerning scoop dehorning. Whereas Coetzee et al. (2012) published that an intravenous administration of meloxicam resulted in significantly (*p* = 0.038) lower SP concentrations in calves after scoop dehorning without LA, compared with control calves [39], Glynn et al. (2013) found no differences in SP concentrations during and after scoop dehorning in calves treated with either a placebo, meloxicam, gabapentin, a combination of meloxicam and gabapentin orally, or flunixin meglumine intravenously; all groups received a cornual nerve block prior to dehorning. In the same study, calves which did not receive any systemic analgesia showed significantly (*p* = 0.02) higher SP concentrations compared with calves treated with a systemic analgesia (137.29 ± 42.97 pg/mL for no analgesia and 63.35 ± 21.25 pg/mL for analgesia, respectively) [41].

No influence of different analgesic regimes of oral meloxicam administration (one or two administrations of meloxicam 24 h apart or placebo treatment on SP concentrations after caustic paste disbudding without LA were published by [44].

### Other

Studies about the evaluation of SP concentrations during painful procedures or conditions other than dehorning or castration were rare (*n* = 4). Theurer et al. (2013) investigated the effect of challenging calves with *Mannheimia haemolytica* and found a significant (*p* < 0.05) interaction between treatment group (challenged compared with control calves) and trial day, with SP concentration being significantly increased in challenged compared with control calves on day 0.5 [48]. Pearson et al. (2019) treated newborn calves following assisted calving with either meloxicam or a placebo and found no differences in SP concentrations between groups over a 24-h period [46]. Studies about painful procedures were published by [18] and [47]. Tschoner et al. (2018) investigated the effect of different analgesic treatments (either meloxicam and a placebo, or meloxicam and metamizole) prior to surgery to correct umbilical hernia under isoflurane anesthesia without LA in calves. Animals treated with both analgesics showed lower SP concentrations during and after umbilical surgery, compared with animals treated with only one analgesic [18]. Another study showed that tail amputation with a rubber band did not have an effect on SP concentration in calves [47].

### Findings: adult cattle

A total of 11 studies evaluating SP concentrations in adult cattle during painful procedures, conditions, or diseases were identified. SP concentrations were evaluated for lameness (36.4%, *n* = 4), diseases (27.3%, *n* = 3), surgeries (27.3%, *n* = 3), and other procedures (9.1%, *n* = 1). Year of publication, authors, grouping of animals, time of blood sampling, extractable SP concentrations, and overall results are presented in Table 3.

**Table 3:** Summary of publication year, reference (Ref.), painful procedures/condition/disease, grouping and age of animals, sampling times, Substance P (SP) concentrations and conclusion of 11 studies using SP for the evaluation of nociception in adult cattle. The administration of local anesthesia (LA) is given in the same column as the grouping of animals. Data was not extractable from 9.1% (*n* = 1) of papers

Year	Ref	Procedure/Disease with/without LA	Grouping	Weight/Age/Lactation	Samplingin Times and Assay Platform	SP Concentrations	Conclusions
**Lameness**							
**2015**	[49]	Oligofructose induced-lameness	- Treatment (13 g/kg BW oligofructose orally, *n* = 6) - Control (*n* = 6) - no LA	250 to 300 kg	- 48 and 24 h before induction of lameness - 6, 12, 24, 36, and 48 h after induction of lameness - ELISA	Mean plasma SP concentrations Control 0.26 to 0.42 ng/mL 12 h after lameness induction: Treatment group 2.20 ± 0.47 ng/mL	Mean plasma SP concentrations increased significantly (*p* < 0.05) 6 h after lameness was induced (treatment group), with a peak 12 h, and decreasing after 48 h after induction. Plasma SP concentrations differed significantly (*p* < 0.001) at every time point after baseline between treatment and control group
**2019**	[50]	Ampothericin B induced-lameness	- L + F (lameness + flunixin, *n* = 10) - L + P (lameness + placebo, *n* = 10) - S + P (sham + placebo, *n* = 10) - no LA	2^nd^ or 3^rd^ lactation	- 6 h before induction of lameness - 1, 2, 8, 24, 48, 72, 96, and 120 h after lameness induction - RIA	Mean SP concentrations L + P 84.59 pg/mL; 95% CI: 73.12 to 96.05 pg/mL L + F 81.89 pg/mL; 95% CI: 72.16 to 91.62 pg/mL S + P 70.59 pg/mL; 95% CI: 55.72 to 85.46 pg/mL	The L + P group had similar SP concentrations to the L + F (topical flunixin meglumine (3.33 mg/kg) for 3 days every 24 h) and S + P group
	[51]	Lameness	- MS 0 (*n* = 25) - MS 1 (*n* = 25) - MS 2 (*n* = 25) - MS 3 (*n* = 25) (on the basis of mobility scoring (MS)) - no LA	1^st^ to 6^th^ lactation, 400 to 500 kg	- 1 sample at last follow up visit - ELISA	Mean SP Concentrations MS 0 0.25 ± 0.09 ng/mL MS 1 0.21 ± 0.13 ng/mL MS 2 0.42 ± 0.12 ng/mL MS 3 0.61 ± 0.12 ng/mL	The mean SP concentrations increased linearly with the increase of MS score. Animals in M3 showed a significant (*p* = 0.000043) increase in SP concentrations compared with MS 0 animals
**2020**	[52]	Ampothericin B induced-lameness	- LAME + FLU (flunixin, *n* = 12) - LAME + MEL (meloxicam, n = 12) - LAME + PLBO (placebo, *n* = 12) - SHAM + PLBO (not lame and placebo, *n* = 12) - no LA		- 24 h before induction of lameness - 0, 2, 8, 24, 48, 72, 96 and 120 h after induction of lameness - RIA	Log LSM SP concentrations LAME + MEL 2.03 pg/mL (95% CI: 1.93, 2.14 pg/ mL) LAME + FLU 2.00 pg/mL (95% CI: 1.90, 2.11 pg/mL) LAME + PBLO 1.98 pg/mL (95% CI: 1.88, 2.09 pg/mL SHAM + PBLO 2.07 pg/mL (95% CI: 1.97, 2.17 pg/ mL	There were no differences between treatments (flunixin meglumine at 2.2 mg/kg BW IV, Meloxicam at1 mg/kg BW orally, or a placebo 2 × every 24 h) or over time for any of the investigated time points
**Diseases**							
**2018**	[53]	Clinical Metritis	- CM (Clinical metritis, *n* = 70) - NO-CM (no clinical metritis, *n* = 88) - no LA		- Day 1 - RIA	Circulating SP Concentrations CM cows 41.15 ± 5.38 pg/mL NO-CM cows 37.73 ± 5.41 pg/mL	Circulating SP concentrations were significantly (*p* = 0.01) higher in CM compared with NO-CM cows
	[54]	Intrapartum Uterine Torsion	- Intrapartum uterine torsion (*n* = 20) - Healthy controls (*n* = 36) - Intrapartum without uterine torsion (*n* = 15) - no LA		- Day 1 - ELISA	Serum SP concentrations Control 37.9 ± 10.5 pg/mL Intrapartum (no uterine torsion) 49.6 ± 14.5 pg/mL Intrapartum (uterine torsion) 32.8 ± 14.1 pg/mL	The SP concentrations were higher in intrapartum cows compared with cows with uterine torsion; also, SP concentrations were significantly (*p* < 0.01) higher in intrapartum compared with healthy cows
**2020**	[55]	Parturition	PRIM (pimiparous, *n* = 47) and MULT (multiparous, *n* = 105), also EUT (eutocia) and DYS (dystocia) divided into the following treatment groups: - ASP (acetylsalicylic acid, *n* = 76, including 38 DYS and 38 EUT) - PLC (placebo, *n* = 76, including 38 DYS and 38 EUT) categorized as - NO-EVT (no disease) - SI-EVT (single disease) - MU-EVT (multiple diseases) - no LA		- 12, 24, 36, and 48 h before parturition (before each treatment administration (4 consecutive treatments at 12 h interval with either acetylsalicylic acid (100 mg/kg orally) or a placebo)—168 ± 72 h after parturition - RIA	Circulating SP Concentrations ASP 56.76 pg/mL, 95% CI: 55.16–58.41 PLC 55.95 pg/mL, 95% CI: 54.36–57.57 At 168 ± 72 h after parturition DYS 64.99 pg/mL, 95% CI: 62.08–68.06 EUT 60.33 pg/mL, 95% CI: 57.65–63.15 PRIM 57.62 pg/mL, 95% CI: 55.62–59.68 MULT 55.11 pg/mL, 95% CI: 53.83–56.42	There was no difference in circulating SP concentrations between both treatments. SP concentrations increased after parturition with the highest levels at 168 h. An interaction (*p* = 0.07) between calving ease and hour after calving was observed; DYS cows showed higher concentrations of SP at 168 ± 72 compared with EUT (*p* = 0.02), and PRIM cows showed higher circulating SP concentrations compared with MULT cows (*p* = 0.04). There was no difference in SP concentrations between animals with a different number of clinical disease events
**Surgeries**							
**2012**	[56]	Electroejaculation	- EEJ - Probed, no EEJ - Control *n* = 9, each bull receiving each treatment - no LA	14.15 ± 0.14 months, 501.9 ± 14.3 kg	- 60 and 30 min before, treatment - 0 min and immediately after treatment - 10, 20, 30, 45, 60, 75, 90, and 120 min after treatment - ELISA	MEAN and SEM plasma SP Concentrations Controll Bulls 93.4 ± 17.2 pg/mL Probed Bulls 79.1 ± 17.2 pg/mL Bulls after Electroejaculation 77.2 ± 17.2 pg/mL	Mean plasma SP concentrations were not different between groups. An effect of time (*p* < 0.0001) could be observed, but no effect of treatment. Also, there was no interaction of treatment and time on SP concentrations
**2020**	[57]	Ovariectomy	- PALP (sham procedure, *n* = 14) - SPAY (ovariectomy, *n* = 15) - BXKM (spay + NSAID, *n* = 15; Combination of butorphanol (0.01 mg/kg BW), xylazine (0.02 mg/kg BW), and ketamine (0.04 mg/kg BW) IM 5 min pre OP and oral meloxicam (1 mg/kg) immediately before surgery - no LA	322 ± 27.0 kg	- D-1 - D0 (at time of procedure) - 1, 2, and 4 h after procedure - Day 1, 2, 4, and 7 after procedure - Competitive Immunoassay	LSM plasma SP Concentrations PALP 78.7 pg/mL SPAY 79.8 pg/mL BXKM 78.6 pg/mL	Regarding SP concentrations, there was no treatment or treatment x interaction effect between groups
	[58]	Endoscopic Abomasopey	- CON (placebo, *n* = 14) - XYL (xylazine, *n* = 14) - local infiltration of skin with 2% procain hydrochloride for both groups	6.0 ± 2.0 years, 662.3 ± 110.7 kg	-180 min (Baseline) before surgery - at the start of surgery - 15, 30, 45, 60, 90, 120, and 180 min after start of the surgery - 24 h after the start of the surgery - ELISA	Mean plasma SP Concentrations Baseline Values CON 555.37 ± 252.77 pg/mL XYL 490.60 ± 219.62 pg/mL	There was no significant difference between plasma SP concentrations between CON and XYL (0.02 mg xylazine IV 15 min before the start of the surgery) at any time point
**Other**							
**2014**	[59]	Long distance transportation	- MEL (meloxicam) - PLACEBO - no LA	15 to 17 months, 201 to 465 kg	Baseline at Time 0, immediately before treatment and 24 and 144 h after baseline sampling - RIA	No extractable numerical data	SP concentrations increased significantly (*p* < 0.0026) after transportation. There was no treatment, or treatment x time interaction, as well as no association between MEL (Meloxicam, at 1 mg/kg BW orally) and SP

*BW* Bodyweight, *IV* Intravenously, *OP* Surgery, *SC* Subcutaneously, *RIA* Radioimmunoassay, *ELISA* Enzyme Linked Immunosorbent Essay

### Lameness

Three studies investigated SP concentrations in adult cattle after experimentally induced lameness (either with Oligofructose, *n* = 1 [49], or Amphotericin B, *n* = 2 [50, 52]). In each of these three studies, grouping and treatment of animals was heterogenous. Bustamante et al. (2015) showed that mean plasma SP concentrations increased significantly (*p* < 0.05) 6 h after induction of lameness with oligofructose, with a peak 12 h after the lameness induction (2.20 ± 0.47 mg/mL). Significant differences (*p* < 0.001) were found at each time point after baseline sampling between control and treatment group [49]. Kleinhenz et al. (2019) and Warren et al. (2021) investigated the effect of different NSAIDs on SP concentrations in cattle with Amphotericin B induced lameness and both stated that there were no significant differences in SP concentrations between animals treated with either a NSAID or a placebo [50, 52].

Only one study compared SP concentrations in a population of cattle with different mobility scores (MS, MS 0 being not lame, to MS 3 being severely lame) and stated that mean SP concentrations increased linearly with the mobility score. Animals with a MS 3 showed significantly (*p* = 0.000043) higher SP concentrations compared with MS 0 (0.61 ± 0.12 ng/mL and 0.25 ± 0.09 ng/mL, respectively) [51].

### Diseases and conditions

Studies describing SP concentrations during painful conditions and diseases were limited to clinical Metritis (*n* = 1), parturition (*n* = 1), and uterine torsion (*n* = 1). Out of these, two studies were part of one larger trial [53, 55]. In 2018, Barragan et al. (2018) compared circulation SP concentrations of cows with or without clinical metritis (diagnosed on day 7 ± 3 after parturition). Cows with clinical metritis had significantly (*p* = 0.01) higher circulation SP concentrations compared with sound animals (41.15 ± 5.38 pg/mL and 37.73 ± 5.41 pg/mL, respectively) [53]. In a follow up paper, the authors found no difference in circulation SP concentrations between animals treated with 100 mg/kg acetylsalicylic acid at a 12-h interval for four times after parturition, compared with animals treated with a placebo. The SP concentrations increased, with a peak at 168 h after parturition. Cows suffering from dystocia had significantly (*p* = 0.01) higher SP concentrations at 168 ± 72 h compared with cows with eutocia; also, primiparous cows showed significantly (*p* = 0.04) higher circulation SP concentrations than multiparous cows [55].

Regarding uterine torsion, serum SP concentrations were significantly (*p* < 0.01) higher in cows during parturition compared with cows with uterine torsion (49.6 ± 14.5 pg/mL and 32.8 ± 14.1 pg/mL). Healthy control cows had significantly (p < 0.01) lower SP concentrations than intrapartum cows (37.9 ± 10.5 pg/mL and 49.6 ± 14.5 pg/mL, respectively) [54].

### Surgeries

SP as a biomarker for pain during surgeries has not been used extensively in adult cattle. Whitlock et al. (2012) evaluated SP concentrations following electroejaculation and found that mean plasma SP concentrations was not different between control (93 ± 17.2 pg/mL), probed (79.1 ± 17.2 pg/mL) and electroejaculated (77.2 ± 17.2 pg/mL) bulls [56]. Another study showed that mean plasma SP concentrations did not differ between female cattle either subjected to ovariectomy following administration of butorphanol, xylazine, and ketamine, ovariectomy without the administration of any analgesia, or palpation only (78.6 pg/mL, 79.8 pg/mL, and 78.7 pg/mL, respectively). Tschoner et al. (2020) investigated the effect of an administration of 0.02 mg/kg BW xylazine or the equivalent amount of 0.9% saline intravenously before laparoscopic abomasopexy following local and systemic analgesia on SP concentrations in cattle and found no differences during and after the surgery between both groups [58].

### Other

One study described the effect of long-distance transporting (16 h, approximately 1.316 km) on plasma SP concentrations in beef steers, and the effect of the administration of a NSAID (meloxicam) on plasma SP concentrations. The plasma SP concentrations increased significantly (*p* < 0.0026) after transportation, but there was no effect of treatment with meloxicam on the SP concentrations [59].

## Discussion

### Findings of the systematic review

The objective of the present study was to give an overview of SP concentrations during and after painful procedures and conditions in calves and cattle. Our aim was to present the different SP concentrations evaluated in the blood plasma for surgeries, procedures, conditions, and diseases, and perform a meta-analysis, if possible. Additionally, we wanted to quantify the existing body of research, also highlighting potential areas where knowledge could and should be increased.

The manageable number of articles extracted from the data bases (*n* = 236) and the small number of references which could be included in this systematic review (*n* = 36) provides evidence that research about SP to evaluate pain in cattle is rare. Even with the number of 36 references, none could be included into a meta-analysis, as study design, grouping, and procedures were too heterogenous. Only a small number of studies compared painful conditions with sham or no intervention control groups, such as [8] for castration and [47] for tail docking in calves, or [51] for lameness in cattle. Most studies used different biomarkers for pain, including SP, to evaluate the effect of different analgesic regimes and the different routes of application (oral, intravenously, subcutaneously) of these analgesics on painful surgeries and procedures. Therefore, the evaluation of SP was not the main focus of these studies, and basic research work is missing. Another problem was the style in which p-values were presented; not all papers presented p-values as accurate numbers, which might be related to the guidelines of the different publishing journals.

Results of studies were heterogenous, especially for dehorning procedures. Whereas Allen et al. (2013) showed that the administration of meloxicam results in significantly lower SP concentrations after cautery dehorning [40], other studies found no effect of systemic analgesics on SP concentrations after different methods of dehorning [21, 42, 43]. Some authors [21, 40, 42] used cornual nerve blocks for local anesthesia of the tissue, whereas some [43] did not work with any local anesthesia. Studies have shown that pre-emptive analgesia prevents the onset of nociception [60, 61]. The administration of multimodal pain management (which is a combination of sedatives, local anesthesia, and nonsteroidal anti-inflammatory drugs) is recommended prior to a painful procedure [61–63]. Different combinations of sedatives, and/or local anesthetics and nonsteroidal anti-inflammatory drugs throughout the studies could explain the inconsistency of SP concentrations in these studies. However, other factors need to be considered, as some studies found no difference in SP concentrations in animals only treated with systemic and no local anesthesia [20, 43].

To this day, no studies describing the SP concentrations in healthy, untreated, and not stressed adult cattle or calves have been described to evaluate physiological ranges of SP concentrations in cattle – although studies showed that SP concentrations differed significantly by age, with 6-months-old calves showing higher concentrations than 8-week-old calves [19]. As there was no consistency among age groups of animals included in studies, SP concentrations cannot be compared easily. Also, gender seems to have an influence on SP; Stock et al. (2016) observed that that SP concentrations were higher in female compared with male calves [42]. In calves, male and female animals were used for the different studies, which, again, makes comparison of concentrations difficult. Even within the same gender and age group, high between- and within-calf variations were found [8]. SP also seems to vary depending on temperament. Kasimanickam et al. (2019) showed that SP concentrations were significantly (*p* < 0.05) higher in excitable compared with calm female cattle prior to weaning and at breeding [64].

Another problem with the present data was that not all references offered numerical data. Some studies only presented graphical data [40], some studies none at all [47]. However, even if data could be extracted, processing of samples for the determination of SP was different throughout the studies, making a comparison of SP concentrations hard. Previous research showed that the temperature blood samples are kept at until further processing, and use of different enzyme inhibitors influence the SP concentrations in the blood plasma [65]. Numerous biological processes can affect the SP concentrations in blood samples after harvesting of blood; therefore, samples should be processed with the same time between collection and harvesting for all samples, and kept on ice until further processing [65]. As this information is not given in all references, and vary throughout the studies, SP concentrations may have been affected by this.

In human medicine, extensive research about SP has been done [13, 14, 16]. The first study describing SP in cattle included in this systematic review dates from 2008. Studies concentrating on pain research in cattle have been neglected for a long period of time, compared with pain research in companion animals and horses – only in the last years did researchers focus their work on pain and pain management in cattle [3, 66]. Pain scoring systems for cattle have been established [6, 67] and the public concern with the welfare of dairy and beef cattle has been raised [68]. The increased interest in pain management in cattle might have resulted in the search for a new and objective biomarker for pain, such as SP. The largest number of studies about SP was published in 2018. However, even if SP is a promising tool to differentiate between stress (caused by e.g. handling) and distress associated with nociception [8], SP has not yet achieved the status of an objective biomarker for nociception which can be used exclusively and without other parameters. Until now, it is recommended to assay SP in combination with cortisol to identify if SP is released due to nociception or stress [44]. Nearly all the references included in this systematic review do not use SP exclusively for the evaluation of nociception, but in combination with other subjective [47, 58] or objective [43, 49, 69] parameters. Other disadvantages of the use of SP have been reviewed recently [25], and include the limited use in the field practice due to the necessary processing of the samples after harvesting of the blood, analysis which can only be done with ELISA [47, 49, 58] or radio immunoassay kits [20, 31, 59], and high costs for the analysis with ELISA kits, e.g. 398,00 Euros/96 wells (Enzo, Enzo Life Sciences GmbH, Lörrach, Germany [70]). Also, the varying study results, as well as the high between- and within-calf variations [8] of SP concentrations might limit the use of SP as an everyday tool for pain assessment.

The largest number of studies was conducted on the effect of different analgesic effects on dehorning and castration in calves – these are common husbandry procedures, especially in the USA [24, 39], where most studies was performed. In the USA, no drugs are federally approved for pain mitigation during these procedures [71], and they are often performed without the use of analgesia [72, 73]. As castration and dehorning are necessary due to e.g. facility design and provision of human safety, and minimizing the pain the animals are experiencing is important [71], the high number of studies concentrating on the effects of analgesics during castration or dehorning can be explained. Also, a recent survey about the attitudes of veterinarians and producers regarding the use of analgesia in beef cattle showed that analgesia was used more frequently in cattle with increased age, regardless of the procedure or disease, and most frequently or always for abdominal surgery, dehorning, lameness, or pneumonia, regardless of the age of the animal [74].

Studies evaluating the effect of different analgesics on animals undergoing painful procedures are necessary, and veterinarians benefit from these studies by receiving guidelines how to improve animal welfare. However, little work has been done in the area of basic research work about the suitability of SP as a biomarker for pain in cattle so far. Studies in human medicine showed that SP plays a role in the activation of the immune system, chemotaxis of granulocytes, and migration of cells to the location of inflamed tissue [16, 75]. SP concentrations increase during an inflammatory process [16, 75, 76]. The same can be said for conditions of emotional stress [77]. In cases of depressions and states of anxiety, the neurotransmission of SP is impaired [78, 79]. Therefore, states of stress and inflammation in cattle could influence the SP concentrations in the blood plasma; however, no explicit research work to answer these questions has been done to this day, which is one of the great limitations of using SP as a biomarker for pain in cattle. Also, no basic values or reference ranges have been established yet, which somehow complicated the comparison of SP concentrations evaluated in different studies.

### Methodology and limitations

This systematic review was done following the PRISMA guidelines [27] to reduce the possible risk of bias due to the analysis and the study selection process. As the exact study type has not been determined when the research work for this systematic review was started, and it was unclear whether a meta-analysis could be performed, a pre-specified protocol of this systematic review was not registered, as has been described for other systematic reviews [28]. Also, registration via PROSPERO is only possible for systematic reviews conducted in human medicine/research. To assure a systematic review process, the authors defined an agenda which was followed to select the studies included in this systematic review. Titles and abstracts were screened independently by two authors to reduce the risk of bias, and full-text screening was done following previously specified guidelines. To assess if data were eligible for a meta-analysis, data were discussed with a statistician as described [28].

### Risk of bias

We used three search engines, to try to not miss any relevant papers or references; using more than one search engine should have reduced the possibility of missing papers. As titles and abstracts were included in the search for keywords, it is unlikely that a large number of papers was not found. Apart from 4 abstracts not being accessible, and 2 abstracts being excluded due to not being in English, all papers which were included in the full-text screening could be assessed. Omitting studies due to language barriers can negatively impact the outcome of a systematic review. However, for this review only two studies could not be included due to this reason – therefore, a bias through limited access should be excluded.

The references we included in this systematic review originate predominantly from the USA and Canada, with only a few studies from other countries. As we evaluated a laboratory parameter, and analysis was done similarly in most studies, the studies included in this systematic review should be representative for other countries as well.

In nearly all the references, funding information was provided, either for the study itself or the authors positions. As published results included studies observing both a positive [10, 39, 40] as well as no [20, 42, 43] effect of NSAIDs on SP concentrations during different procedures, a publication bias due to the influence of the source funding the studies seems unlikely – especially as funding mostly came from animal welfare organizations, national research councils, or universities.

## Conclusion

Pain in cattle is a major welfare problem, and the need for objective parameters to assess pain is evident. Our work shows that results of research work about SP as a pain biomarker in cattle is heterogenous, and concentrations differ throughout studies and study designs. Basic research work is needed to evaluate if SP concentrations are largely influenced by nociception, or also by stress and states of inflammation. Also, reference ranges should be established to make comparison of concentrations between sound animals, and animals in pain, easier. Therefore, this systematic review should aid researchers with their decision on objectives and study design for future research. Future studies on the suitability of SP as a biomarker for pain in cattle can improve the pain management and welfare of adult cattle and calves. 

## Supplementary Information

**Additional file 1.** PRISMA-P checklist for the systematic review “Substance P concentrations in adult cattle and calves during different painful procedures and conditions – a systematic review” according to Shamseer et al. (2015).

**Additional file 2.** Funding information for 36 references (Ref.) included in the systematic review “Substance P concentrations in adult cattle and calves during different painful procedures and conditions – a systematic review”. If no funding information was retrievable, this is indicated as “none given”.

## Data Availability

The data used in this work is indicated and lies with the author. It can be assessed via the corresponding author.

## Abbreviations

LA: Local anesthesia
NSAID: Non-steroidal anti-inflammatory drug
PRIMA: Preferred Reporting Items for Systematic Reviews and Meta-Analysis
SP: Substance P
USA: United States of America
