# Supplementary material for: Substance P concentrations in the blood plasma and serum of adult cattle and calves during different painful procedures and conditions – a systematic review
Source: BMC Vet Res. 2022 Jun 18;18:232. doi: 10.1186/s12917-022-03304-6 (PMC9206354; doi:10.1186/s12917-022-03304-6)
Supplement: Supplementary file 1 — Additional file 1. PRISMA-P checklist for the systematic review “Substance P concentrations in adult cattle and calves during different painful procedures and conditions – a systematic review” according to Shamseer et al. (2015). [file 12917_2022_3304_MOESM1_ESM.docx]

**Additional file 1:** PRISMA-P checklist for the systematic review “Substance P concentrations in adult cattle and calves during different painful procedures and conditions – a systematic review” according to Shamseer et al. (2015).

| Section and Topic | Item Nr. | Checklist Item |
| --- | --- | --- |
| **Administrative Information** | | |
| Title | | |
| Identification | 1a | Identify the report as a protocol of a systematic review  - Protocol for a systematic review to evaluate substance P concentrations in cattle during painful procedures and conditions, such as diseases |
| Update | 1b | If the protocol is for an update of a previous systematic review, identify as such  - This is the first attempt for a systematic review covering the above-mentioned content, and no update of a previously conducted systematic review. |
| Registration | 2 | If registered, provide the name of the registry (such as PROSPERO) and registration number  - Not applicable, as according to its website, PROSPERO can only be used to studies in human medicine/human patients. |
| Authors | | |
| Contact | 3a | Provide name, institutional affiliation, e-mail address of all protocol authors; provide physical mailing address of corresponding author  - Corresponding author: Theresa Tschoner  [t.tschoner@lmu.de](mailto:t.tschoner@lmu.de)  Melanie Feist: [M.Feist@lmu.de](mailto:M.Feist@lmu.de)  Author affiliation:  Clinic for Ruminants with Ambulatory and Herd Health Services, Centre for Clinical Veterinary Medicine, LMU Munich, Sonnenstrasse 16, 85764 Oberschleissheim, Germany |
| Contributions | 3b | Describe contributions of protocol authors and identify the guarantor of the review  - TT is the guarantor; TT drafted the manuscript, TT and MF developed the search strategy and the criteria for the selection. TT and MF selected the references for full-text review, TT did the full text review. Both authors read, proved feedback, and approved the final manuscript. |
| Amendments | 4 | If the protocol represents an amendment of a previously completed or published protocol, identify as such and list changes; otherwise, state plan for documenting important protocol amendments  - This protocol doesn’t represent an amendment of a previously completed/published protocol. If amendments of this protocol are necessary, they will be included with the date of the amendment, and a description thereof. |
| Support | | |
| Sources | 5a | Indicate sources of financial or other support for the review  - There was no financial support of this study itself. The study was conducted in the course of the habilitation of TT. Salary was ensured to MF. TT was funded with a 50% Bavarian Gender Equality Fund (BGF). |
| Sponsor | 5b | Provide name for the review funder and/or sponsor  - Not applicable. |
| Role of sponsor or funder | 5c | Describe roles of funder(s), sponsor(s), and/or institution(s), if any, in developing the protocol  - Not applicable. |
| **Introduction** | | |
| Rationale | 6 | Describe the rationale for the review in the context of what is already known  - The evaluation of pain in cattle is a major welfare problem, as cattle are stoic animals and mask their pain. Subjective and objective parameters for the evaluation of pain have been described; among the objective parameters, substance P is described to be very useful to differentiate between stress caused by handling, and distress caused by nociception. However, there is no large number about studies which have been conducted on the evaluation of substance P in cattle, and as substance P concentrations were mostly evaluated during different study settings and painful procedures, comparison of studies is difficult. To this day, no systematic review or meta-analysis about the evaluation of substance P has been published. Therefore, the objective of the present paper was 1) to assess substance P concentrations in adult cattle and calves associated with different (painful) procedures, conditions, and diseases, and 2) to evaluate the literature in form of a systematic review. The aim of this review is to be a contribution to the current knowledge about substance P in cattle by giving an overview of the existing literature concerning the research about this pain parameter, and to identify and outline the areas of lack of knowledge. |
| Objectives | 7 | Provide an explicit statement of the question(s) the review will address with reference to participants, interventions, comparators, and outcomes (PICO)  - The aim of this systematic review is to evaluate the substance P concentrations in adult cattle and calves during and after painful procedures, conditions, and diseases. |
| **Methods** | | |
| Eligibility criteria | 8 | Specify the study characteristics (such as PICO, study design, setting, time frame) and report characteristics (such as years considered, language, publication status) to be used as criteria for eligibility for the review  - If the evaluation of substance P during and after painful procedures, conditions, or diseases is described, all studies published in English or German will be selected. The authors expect mainly experimental studies, but will not excluded any study type, to include as many articles as possible. The authors will define no time frame, the literature search will be performed to the date of the 28^th^ of September 2021. Studies will be included if the following 4 questions can be answered with “yes”:   1. Is the study population either cattle or calves? 2. Is Substance P used as a biomarker for pain/nociception? 3. Are animals undergoing a painful procedure (such as castration, dehorning, …) 4. Is the article peer-reviewed? |
| Information sources | 9 | Describe all intended information sources (such as electronic databases, contact with study authors, trial registers or other grey literature sources) with planned dates of coverage  - The literature search will be conducted with the following data bases: MEDLINE, PubMed, Web of Science. |
| Search strategy | 10 | Present draft of search strategy to be used for at least one electronic database, including planned limits, such that it could be repeated  - The main elements of this review are Cattle, substance P, and Pain; the population search terms will be (cattle OR cow OR calves OR bull OR steer), and the outcome search terms will be ("substance P") and (pain* OR nociception). The code used for all four databases will be:  - (cattle OR cow OR calves OR bull OR steer)  AND  - ("substance P")  AND  - (pain* OR nocicept*) |
| Study records | | |
| Data management | 11a | Describe the mechanism(s) that will be used to manage records and data throughout the review  - We will use Microsoft Ecxel and Endnote to manage the records and data. |
| Selection process | 11b | State the process that will be used for selecting studies (such as two independent reviewers) through each phase of the review (that is, screening, eligibility and inclusion in meta-analysis)  - After deduplication of titles, both authors (TT and MF) will independently screen all titles, and afterwards all abstracts of the articles included after the search and check for the inclusion criteria. Afterwards, all full texts of the included articles will be obtained and screened by TT and will be included in the systematic review if they meet the inclusion criteria (substance P, painful procedure/condition/disease, adult cattle or calves, peer-reviewed). Neither author will be blinded to the title, authors, or journal in which the reference has been published. If full texts can’t be retrieved, the publishing authors will be contacted. |
| Data collection process | 11c | Describe planned method of extracting data from reports (such as piloting forms, done independently, in duplicate), any processes for obtaining and confirming data from investigators  - Data extraction will be done by TT after discussion with MF. The extracted data will include information on authors, publication year and country, age of animals, procedure/condition/disease described, number and timing of substance P samples retrieved, processing of these samples after harvesting, substance P concentrations, influence of intervention on substance P concentrations, and funding information. Data will be extracted and collected using Miscrosoft Excel. |
| Data items | 12 | List and define all variables for which data will be sought (such as PICO items, funding sources), any pre-planned data assumptions and simplifications  - Data extraction will include information on authors, publication year and country, age of animals, procedure/condition/disease described, number and timing of substance P samples retrieved, processing of these samples after harvesting, substance P concentrations, influence of intervention on substance P concentrations, and funding information. Data will be extracted and collected using Miscrosoft Excel. Data and information will be checked for the suitability to perform a meta-analysis. |
| Outcomes and prioritization | 13 | List and define all outcomes for which data will be sought, including prioritization of main and additional outcomes, with rationale  - The primary outcome will be v concentrations in adult cattle and calves, during different procedures/conditions/diseases. Data will be sought for the outcomes substance P, adult cattle or calves, and pain or nociception. |
| Risk of bias in individual studies | 14 | Describe anticipated methods for assessing risk of bias of individual studies, including whether this will be done at the outcome or study level, or both; state how this information will be used in data synthesis  - If a meta-analysis can be done, potential bias across studies will be presented graphically. If not, it will be presented in the sections “results” and “discussion”. |
| Data synthesis | 15a | Describe criteria under which study data will be quantitatively synthesised  - If the studies included in this systematic review are homogenous in study design and comparator, and if they provide statistical information about substance P which can be retrieved from the data, a meta-analysis will be performed. |
|  | 15b | If data are appropriate for quantitative synthesis, describe planned summary measures, methods of handling data and methods of combining data from studies, including any planned exploration of consistency (such as I2, Kendall’s τ)  - If data are appropriate for a quantitative synthesis, Odds ratios (OR) and their 95% confidence intervals will be calculated. |
|  | 15c | Describe any proposed additional analyses (such as sensitivity or subgroup analyses, meta-regression)  - Not planned. |
|  | 15d | If quantitative synthesis is not appropriate, describe the type of summary planned  - If a quantitative synthesis can’t be performed, a verbal summary of the findings of the systematic review will be done, including the evaluation of substance P concentrations during the different procedures/conditions/diseases. |
| Meta-bias(es) | 16 | Specify any planned assessment of meta-bias(es) (such as publication bias across studies, selective reporting within studies)  - If a meta-analysis can be done, potential bias across studies will be presented graphically. If not, it will be presented in the sections “results” and “discussion”. |
| Confidence in cumulative evidence | 17 | Describe how the strength of the body of evidence will be assessed (such as GRADE)  - Studies will be included in the systematic review if the following 4 questions can be answered with “yes”, as described by [29]:   1. Is the study population either cattle or calves? 2. Is Substance P used as a biomarker for pain/nociception? 3. Are animals undergoing a painful procedure (such as castration, dehorning, …) 4. Is the article peer-reviewed?   Apart from that, no other reporting guidelines will be used. |
