# Supplementary material for: Substance P concentrations in the blood plasma and serum of adult cattle and calves during different painful procedures and conditions – a systematic review
Source: BMC Vet Res. 2022 Jun 18;18:232. doi: 10.1186/s12917-022-03304-6 (PMC9206354; doi:10.1186/s12917-022-03304-6)
Supplement: Supplementary file 2 — Additional file 2. Funding information for 36 references (Ref.) included in the systematic review “Substance P concentrations in adult cattle and calves during different painful procedures and conditions – a systematic review”. If no funding information was retrievable, this is indicated as “none given”. [file 12917_2022_3304_MOESM2_ESM.docx]

**Additional file 2:** Funding information for 36 references (**Ref.**) included in the systematic review “Substance P concentrations in adult cattle and calves during different painful procedures and conditions – a systematic review”. If no funding information was retrievable, this is indicated as “none given”.

| **Year** | **Ref.** | **Author** | **Funding** |
| --- | --- | --- | --- |
| **Calves** | | | |
| **Castration** | | | |
| 2008 | [8] | Coetzee et al. | - College of Veterinary Medicine, Kansas State University |
| 2013 | [19] | Dockweiler et al. | - Agriculture and Food Research Initiative Competitive (Grant number 2009-65120-05729) from the USDA National Institute of Food and Agriculture  - National Center for Research Resources (award number T35RR007064 )  - J. F. Coetzee was supported by the Agriculture and Food Research Initiative Competitive (Grant number 2008-35204-19238) from the USDA National Institute of Food and Agriculture  - in part funded by Kansas State University, College of Veterinary Medicine, Departments of Anatomy and Physiology and Clinical Sciences. |
|  | [36] | Repenning et al. | - None given |
| 2014 | [30] | Mintline et al. | - Russell L. Rustici Rangeland and Cattle Research Endowment |
| 2016 | [10] | Olson et al. | - Alberta Livestock and Meat Agency (ALMA, 2011R043R)  - Alberta Veterinary Laboratories. |
| 2017 | [33] | Marti et al. | - Agriculture and Agri-Food Canada and the Beef Cattle Research Council through the Canadian Beef Cattle Industry Science Cluster |
|  | [5] | Meléndez et al. | - Agriculture and Agri-Food Canada and the Beef Cattle Research Council through the Canadian Beef Cattle Industry Science Cluster |
|  | [34] | Meléndez et al. | - Agriculture and Agri-Food Canada and the Beef Cattle Research Council through the Canadian Beef Cattle Industry Science Cluster |
| 2018 | [35] | Meléndez et al. | - Agriculture and Agri-Food Canada and the Beef Cattle Research Council through the Canadian Beef Cattle Industry Science Cluster  - S. Marti was supported by the CERCA program from Generalitat de Catalunya |
|  | [75] | Meléndez et al. | - Agriculture and Agri-Food Canada and the Beef Cattle Research Council through the Canadian Beef Cattle Industry Science Cluster  - S. Marti was partly supported by the CERCA pro- gram from Generalitat de Catalunya |
|  | [20] | Kleinhenz et al. | - Pharmacology Analytical Support Team (PhAST) in the College of Veterinary Medicine at Iowa State University. |
|  | [31] | Park et al. | - Korea Institute of Planning and Evaluation for Technology in Food, Agriculture, Forestry, and Fisheries (IPET) through Advanced Production Technology Development Program  - Ministry of Agriculture, Food, and Rural A!airs (MAFRA) (118016-03-1-SB010). |
| 2019 | [32] | Meléndez et al. | - Agriculture and Agri-Food Canada (http://www.agr.gc.ca/eng/ home/?id=1395690825741) and the Beef Cattle Research Council (http://www.beefresearch.ca/) through the Canadian Beef Cattle Industry Science Cluster (ANH.21.13 AIP-CL01)  - S. Marti was partly supported by the CERCA |
| 2021 | [76] | Bergamasco et al. | - USDA-CSREES NRI (Award No. 2009-65120-05729) |
| **Dehorning** | | | |
| 2012 | [40] | Coetzee et al. | - J. F. Coetzee and R. A. Mosher were supported by USDA- CSREES, Animal Protection (Animal Well-being, NRI Grant # 2008-35204- 19238 and 2009-65120-05729) |
| 2013 | [37] | Allen et al. | - Agriculture and Food Research Initiative Competitive Grantno. 2009-65120-05729 from the USDA National Institute of Food and Agriculture  - J. F. Coetzee was supported by Agriculture and Food Research Initiative Competitive Grant #2008-35204-19238 from the USDA National Institute of Food and Agriculture. |
|  | [41] | Glynn et al. | - None given |
| 2015 | [21] | Stock et al. | - Pharmacology Analytical Support Team (PhAST) in the College of Veterinary Medicine at Iowa State University |
| 2016 | [38] | Stock et al. | - Pharmacology Analytical Support Team (PhAST) in the College of Veterinary Medicine at Iowa State University |
| 2017 | [39] | Kleinhenz et al. | - Pharmacology Analytical Support Team (PhAST) in the College of Veterinary Medicine at Iowa State University |
| 2019 | [42] | Karlen et al. | - University of Wisconsin—River Falls (UWRF) Undergraduate Research, Scholarly, and Creative Activity program, UWRF Falcon Scholars program  - Kraft Heinz Company |
| **Other** | | | |
| 2013 | [43] | Theurer et al. | - Merck Animal Health, Desoto, KS |
| 2018 | [18] | Tschoner et al. | - None given |
| 2019 | [44] | Pearson | - University of Calgary Clinical Research Fund  - Anderson Chisholm Chair in Animal Care and Welfare  - Alberta Agriculture and Forestry  - J. M. Pearson’s stipend support by the University of Calgary Eyes High Scholarship |
| 2020 | [45] | Mayer et al. | - None given |
| **Adult cattle** | | | |
| **Lameness** | | | |
| 2015 | [46] | Bustamante et al. | - project CONICYT/ FONDECYT/INICIACION no. 11121615. |
| 2018 | [49] | Rodriguez et al. | - Project CONICYT/FONDECYT/ INICIACION No 11121615 |
| 2019 | [47] | Kleinhenz et al. | - Pharmacology Analytical Support Team (PhAST) at the College of Veterinary Medicine, Iowa State University |
| 2020 | [48] | Warner et al. | - American Association of Bovine Practitioners Foundation (Ashland, OH). |
| **Diseases** | | | |
| 2018 | [50] | Barragan et al. | - partly funded by the Ohio Dairy Producers Association (Columbus, OH) |
| 2018 | [77] | Sickinger et al. | - Ewald and Hilde Berge Foundation, Germany |
| 2020 | [51] | Barragan et al. | - partly funded by the Ohio Dairy Producers Association (Columbus, OH) |
| **Surgeries** | | | |
| 2012 | [52] | Whitlock et al. | - None given. |
| 2020 | [78] | Lauder et al. | - Canada/Alberta Livestock Research Trust Inc.  - British Columbia Cattlemen’s Association (Beef Cattle Industry Development Fund; Project #520). |
| 2020 | [53] | Tschoner et al. | - None given |
| **Other** | | | |
| 2014 | [54] | Van Engen et al. | - Department of Veterinary Diagnostic and Production Animal Medicine (VDPAM) in the College of Veterinary Medicine at Iowa State University  - J. F. Coetzee was supported by Agriculture and Food Research Initiative Competitive (Grant no. 2013-67015-21332) from the USDA National Institute of Food and Agriculture |
